# Disruption of c-*Kit* Signaling in *Kit*^*W-sh/W-sh*^ Growing Mice Increases Bone Turnover

**DOI:** 10.1038/srep31515

**Published:** 2016-08-16

**Authors:** Sutada Lotinun, Nateetip Krishnamra

**Affiliations:** 1 Department of Physiology and STAR on Craniofacial and Skeletal Disorders, Faculty of Dentistry, Chulalongkorn University, Bangkok, Thailand; 2 Department of Oral Medicine, Infection, and Immunity, Harvard School of Dental Medicine, Boston, MA, USA; 3 Department of Physiology, Faculty of Science, Mahidol University, Bangkok, Thailand

## Abstract

c-Kit tyrosine kinase receptor has been identified as a regulator of bone homeostasis. The *c-Kit* loss-of-function mutations in WBB6F1/J-*Kit*^*W/W-v*^ mice result in low bone mass. However, these mice are sterile and it is unclear whether the observed skeletal phenotype is secondary to a sex hormone deficiency. In contrast, C57BL/6J-*Kit*^*W-sh*^/^*W-sh*^ (*W*^*sh*^*/W*^*sh*^) mice, which carry an inversion mutation affecting the transcriptional regulatory elements of the *c-Kit* gene, are fertile. Here, we showed that *W*^*sh*^*/W*^*sh*^ mice exhibited osteopenia with elevated bone resorption and bone formation at 6- and 9-week-old. The *c-Kit W*^*sh*^ mutation increased osteoclast differentiation, the number of committed osteoprogenitors, alkaline phosphatase activity and mineralization. *c-Kit* was expressed in both osteoclasts and osteoblasts, and *c-Kit* expression was decreased in *W*^*sh*^*/W*^*sh*^osteoclasts, but not osteoblasts, suggesting an indirect effect of *c-Kit* on bone formation. Furthermore, the osteoclast-derived coupling factor Wnt10b mRNA was increased in *W*^*sh*^*/W*^*sh*^ osteoclasts. Conditioned medium from *W*^*sh*^*/W*^*sh*^ osteoclasts had elevated Wnt10b protein levels and induced increased alkaline phosphatase activity and mineralization in osteoblast cultures. Antagonizing Wnt10b signaling with DKK1 or Wnt10b antibody inhibited these effects. Our data suggest that *c-Kit* negatively regulates bone turnover, and disrupted *c-Kit* signaling couples increased bone resorption with bone formation through osteoclast-derived Wnt 10 b.

c-Kit, a receptor tyrosine kinase belonging to the platelet-derived growth factor (PDGF) and the colony-stimulating factor 1 (CSF-1) receptor family, is a product of the gene at the *Dominant White Spotting* (*W*) locus^1^,^2^. The ligand for *c-Kit* is the gene product of the *Steel* (*Sl*) locus and is known as mast cell growth factor, stem cell factor, steel factor, and Kit ligand (KL)^3^,^4^. c*-*Kit and KL are essential for normal development and maintenance of three stem cell populations: germ cells, neural crest–derived melanocytes, and hematopoietic stem cells. c-Kit is present in primordial germ cells, spermatogonia, primordial oocytes, growing oocytes, melanocytes^5^, mast cells^6^, and osteoclasts^7^. Homozygotes carrying mutations at the *W* and *Sl* loci are erythrocyte- and mast cell-deficient, infertile, and lack pigmented coats^8^.

Several naturally occurring loss-of-function mutations of *c-Kit* have been identified in mice and humans. The *W* mutation is a null mutation causing deletion of the transmembrane domain of the c-Kit receptor, while *W*^*v*^ is a point mutation in the kinase domain of the receptor resulting in impaired receptor activity^9^. Cells expressing the *W*^*v*^ mutation do not respond to KL in proliferation and apoptosis assays, presumably due to the inability of the receptor to initiate signal transduction^10^,^11^,^12^. *W-sash* (*W*^*sh*^), an allele of *W,* is an inversion mutation upstream of the *c-Kit* promoter region affecting a key regulatory element, resulting in cell-type-specific altered expression of the gene^13^,^14^,^15^,^16^. The *W*^*sh*^ mutation arises spontaneously from crossing two inbred strains of C3H/HeH and 101/H mice.

The role of KL/c-Kit signaling in the regulation of bone metabolism has been studied *in vitro* and *in vivo*. The human osteoblast-like cell line Saos-2 expresses KL on its cell surface, whereas the osteoclast progenitor-like cell line FLG 29.1 expresses c-Kit^7^. Based on these studies, it was concluded that the c-Kit receptor mediates cell-to-cell interactions between osteoclasts and osteoblasts/stromal cells through membrane-bound KL. Previous studies have identified skeletal changes in *Sl/Sl*^*d*^ mutants lacking the transmembrane form of KL but had normal c-Kit receptors^17^. Deletion of membrane-bound KL induces osteopenia. The negative bone balance observed in these mice was primarily due to increased osteoclast surface. It has been shown that 14-week-old female WBB6F1/J-*Kit*^*W/W-v*^ (*W/W*^*v*^) mice carrying a compound *c-Kit* mutation were osteopenic^18^. However, these mice were infertile due to a lack of germ cells in the ovary and had reduced estrogen and progesterone levels, leading to increased FSH level^19^. It is unclear whether the observed skeletal phenotype in *W/W*^*v*^ mice resulted from cell-autonomous effects in osteoclasts or was a consequence of changes in sex hormone levels. In the present study, we focused on the skeletal phenotype of C57BL/6J-*Kit*^*W-sh/*^^*W-sh*^ (*W*^*sh*^*/W*^*sh*^) mice that were fully fertile and determined the mechanism by which altered *c-Kit* signaling affected bone turnover. Our data indicated that the *c-Kit W*^*sh*^ mutation resulted in decreased cancellous bone volume with an increase in bone resorption and bone formation in growing mice. Calvarial osteoblasts derived from *W*^*sh*^*/W*^*sh*^mice showed an increase in osteoblast precursors, alkaline phosphatase (ALP) activity and mineralization *in vitro*. Moreover, the RANKL/OPG ratio was increased in osteoblasts derived from these mice, leading to increased number of osteoclasts in c-*Kit* mutants. Quantitative real-time PCR (qPCR) indicated that *c-Kit* expression was decreased in *W*^*sh*^*/W*^*sh*^bone marrow macrophage (BMM) and osteoclasts but not osteoblasts, suggesting that increased bone formation in *W*^*sh*^*/W*^*sh*^ mice was not an osteoblasts intrinsic effect. Conditioned medium derived from *W*^*sh*^*/W*^*sh*^ osteoclasts contained increased levels of the osteoclast-derived coupling factor Wnt10b, and enhanced ALP activity and mineralization by osteoblasts. Blocking Wnt10b activity inhibited these effects. These findings demonstrate that c-*Kit* regulates bone turnover by suppressing osteoblast and osteoclast differentiation. Thus, *c-Kit* mutation increased bone formation by increasing the generation of osteoclast-derived coupling factor Wnt10b.

## Results

### *W/W* ^*v*^ mice exhibited osteopenic phenotype

**Figure 1:**
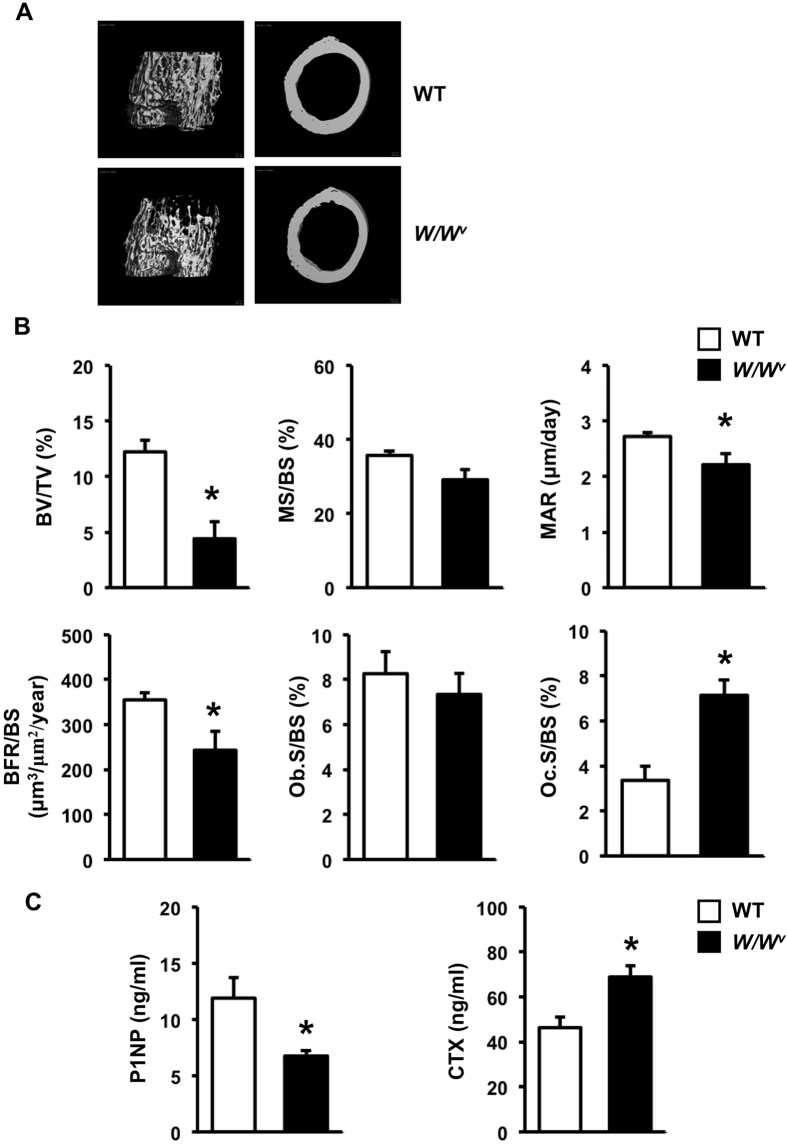
Six-week-old male *W/W*^*v*^ mice are osteopenic. (**A**) Representative μCT images of cancellous (left) and cortical bone (right) from femora of WT and *W/W*^*v*^ mice. (**B**) Histomorphometric analysis of cancellous bone in tibiae. (**C**) Serum concentration of P1NP and CTX (ng/ml). Results are mean ± SEM. *p < 0.05 versus WT.

Previous studies indicated that both male and female *W/W*^*v*^ mice are infertile^19^,^20^. To determine whether a sex hormone deficiency contributed to changes in the skeletal phenotype of growing *W/W*^*v*^ mice, we first analyzed the skeletal phenotype of 6-week-old *W/W*^*v*^ mice. Theses mutants were smaller compared with wild type (WT) littermates and their body weight was 20% less (24.50 ± 0.88 g in WT vs 19.49 ± 0.42 g in *W/W*^*v*^ mice, *p* < 0.05). μCT analysis showed a decrease in cancellous bone volume, trabecular thickness, trabecular number and connectivity density with a concomitant increase in trabecular separation (Fig. 1A and Supplementary Table S1). Cross-sectional volume, cortical volume, and cortical thickness were also decreased in *W/W*^*v*^ mice compared with controls. Histomorphometric analysis confirmed a significant decrease in cancellous bone volume (Fig. 1B). The cancellous bone was less dense and thinner in *W/W*^*v*^ mice with decreased trabecular number and thickness and increased trabecular separation (data not shown). Bone formation was reduced due to a slight decrease in mineralizing surface per bone surface (*p* = 0.052) and a significant decrease in mineral apposition rate. Although osteoblast surface per bone surface was not changed in the mutants, osteoclast surface per bone surface was significantly increased. Therefore, the reduction in bone volume in the mutants was the result of decreased bone formation and increased bone resorption. As expected, *W/W*^*v*^ mice had decreased serum P1NP and increased serum CTX, confirming uncoupled bone turnover (Fig. 1C). Seminal vesicle weight, an index of androgen deficiency, was lower in *W/W*^*v*^ mice (0.122 ± 0.009 g in WT vs 0.071 ± 0.005 g in *W/W*^*v*^ mice, *p* < 0.05). Serum testosterone was significantly decreased in *W/W*^*v*^ mice (2.21 ± 0.30 ng/ml) compared with WT controls (5.02 ± 1.19 ng/ml).

### *Growing W* ^*sh*^ */W* ^*sh*^ mice are osteopenic

**Figure 2:**
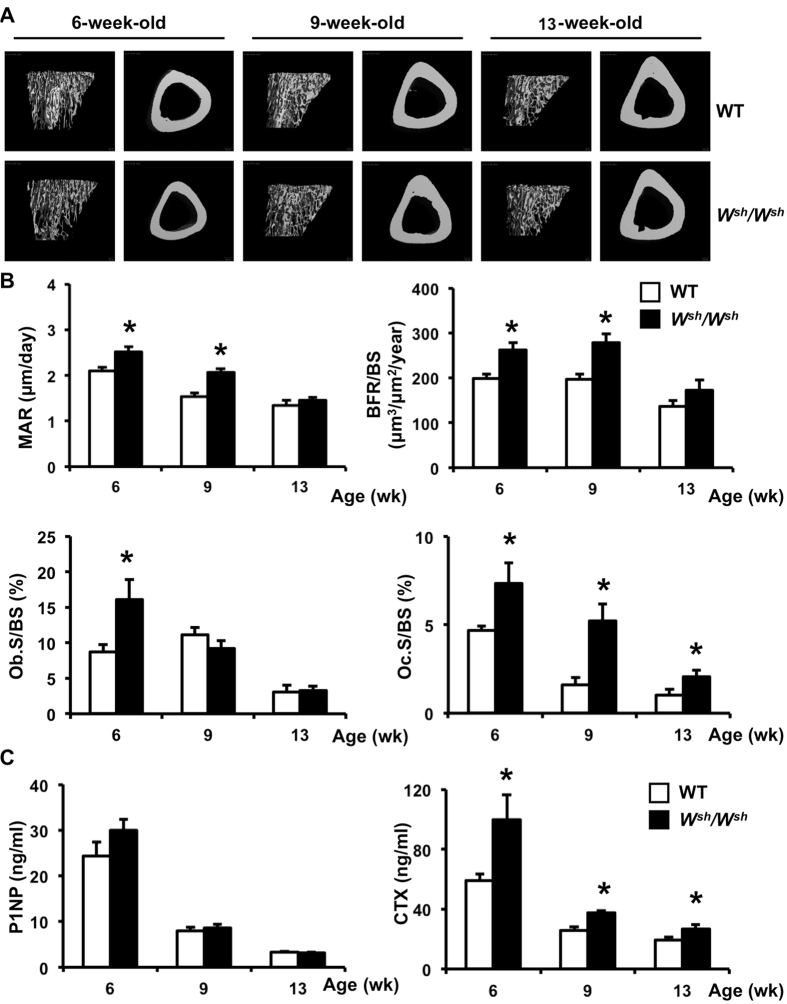
Mutation of c-*Kit* increases bone formation and bone resorption in growing male mice. (**A**) Representative μCT images of cancellous (left) and cortical bone (right) from tibiae of 6-, 9-, and 13-week-old WT and *W*^*sh*^*/W*^*sh*^ mice. (**B**) Histomorphometric analysis of cancellous bone in tibiae. (**C**) Serum concentration of P1NP and CTX (ng/ml). Results are mean ± SEM. *p < 0.05 versus WT.

To eliminate the possible effect of sex hormones on the skeletal phenotype in *c-Kit* mutants, we analyzed the skeletal phenotype of male *W*^*sh*^*/W*^*sh*^ mice. The body weight of the mutants and WT were similar (data not shown). μCT analysis of the cortical bone indicated that *c-Kit* mutation resulted in a significant decrease in total cross sectional volume, cortical volume, and marrow volume at 6, but not 9 and 13 weeks of age (Fig. 2A and Supplementary Table S2). A significant decrease in cancellous bone volume, trabecular number and connectivity density with a concomitant increase in trabecular separation was observed in 6- and 9-week-old *W*^*sh*^*/W*^*sh*^ mice. Unlike the *W/W*^*v*^ mice, seminal vesicle weight was similar in *W*^*sh*^*/W*^*sh*^ mice and WT controls (data not shown). The serum testosterone levels in 6-week-old mice (6.05 ± 1.08 ng/ml in WT vs 5.84 ± 1.44 ng/ml in *W*^*sh*^*/W*^*sh*^ mice, NS) confirmed that male *W*^*sh*^*/W*^*sh*^ mice are not androgen deficient.

### *W* ^*sh*^ mutation increases bone formation and bone resorption in growing mice

Histomorphometric analysis of the tibiae confirmed a decrease in cancellous bone volume at 6 weeks of age in *W*^*sh*^*/W*^*sh*^ mice (Supplementary Table S3). Although mineralizing surface was not affected, mineral apposition rate was higher in 6- and 9-week-old *W*^*sh*^*/W*^*sh*^ mice, leading to increased bone formation rate (Fig. 2B and Supplementary Table S3). Indices of bone formation; osteoblast surface per bone surface, osteoid surface per bone surface, osteoid volume per tissue volume, and osteoid thickness, were markedly increased at 6 weeks of age. A trend toward an increase in serum P1NP was also observed (Fig. 2C). However, the skeletal phenotype was milder in older mice. There was no statistical significant difference between control and mutant mice in all indices of bone formation at 13 weeks of age. In contrast, osteoclast surface per bone surface was dramatically increased compared with age-matched controls in all age groups. As shown in Fig. 2C, increased bone resorption in the mutants was confirmed by increased serum CTX levels.

We then examined the skeletal phenotype of 6 weeks old female mice. Similar to male *W*^*sh*^*/W*^*sh*^ mice, female mice had increased bone turnover. As shown in Supplementary Table S4, mineral apposition rate was higher in female *W*^*sh*^*/W*^*sh*^ mice compared with WT, leading to an increase in bone formation rate expressed per bone surface and bone volume. Osteoblast surface per bone surface, osteoblast number per tissue area and osteoblast number per bone perimeter were significantly increased in 6-week-old *W*^*sh*^*/W*^*sh*^ mice. Osteoclast surface per bone surface, osteoclast number per tissue area and osteoclast number per bone perimeter were also increased. The magnitude of change in bone formation rate was higher in female (40–57%) compared with male mice (30–37%). Therefore, there was no net change in bone volume in female mice. Male *W*^*sh*^*/W*^*sh*^ and their controls were selected for further investigation.

**Figure 3:**
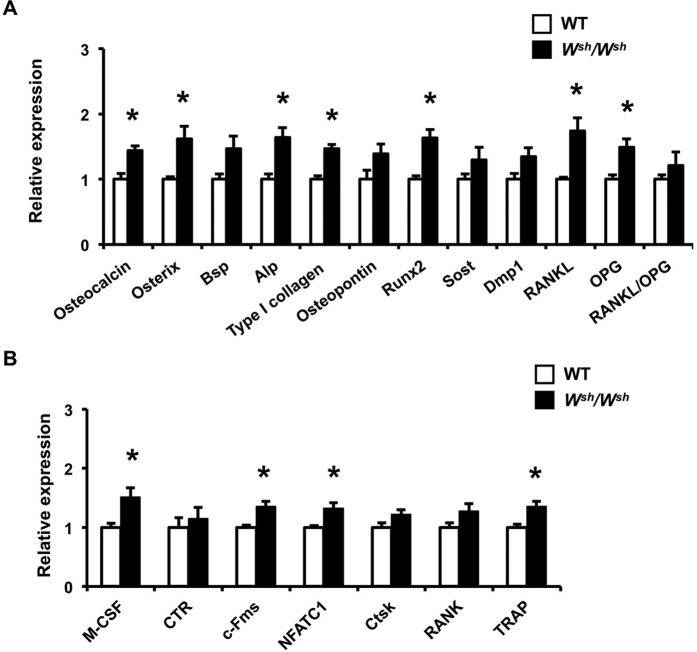
Osteoblast and osteoclast marker genes are upregulated in the distal femoral metaphysis of 6-week-old male WT and *W*^*sh*^*/W*^*sh*^ mice. (**A**) qPCR analysis of osteoblast marker genes. (**B**) qPCR analysis of osteoclast marker genes. Results are mean ± SEM. *p < 0.05 versus WT.

Osteoblast and osteoclast marker gene expression was examined in 6-weeks-old male *W*^*sh*^*/W*^*sh*^mice and their controls. As shown in Fig. 3A, qPCR indicated that *c-Kit* mutation increased the expression of several osteoblast marker genes in femora including osteocalcin, Osterix, ALP, type I collagen and Runx2. The mRNA levels of both RANKL and OPG were increased therefore the RANKL/OPG ratio was not significantly changed. Expression profiling of osteoclast target genes showed increased expression of M-CSF, c-Fms, NFATC1 and TRAP in 6-week-old *W*^*sh*^*/W*^*sh*^ mice (Fig. 3B). These data suggest that the increased bone turnover observed in 6-week-old *W*^*sh*^*/W*^*sh*^ mice is likely to be due to increased bone formation and bone resorption *in vivo*.

### *c-Kit* mutation increases osteoclast differentiation and precursors

**Figure 4:**
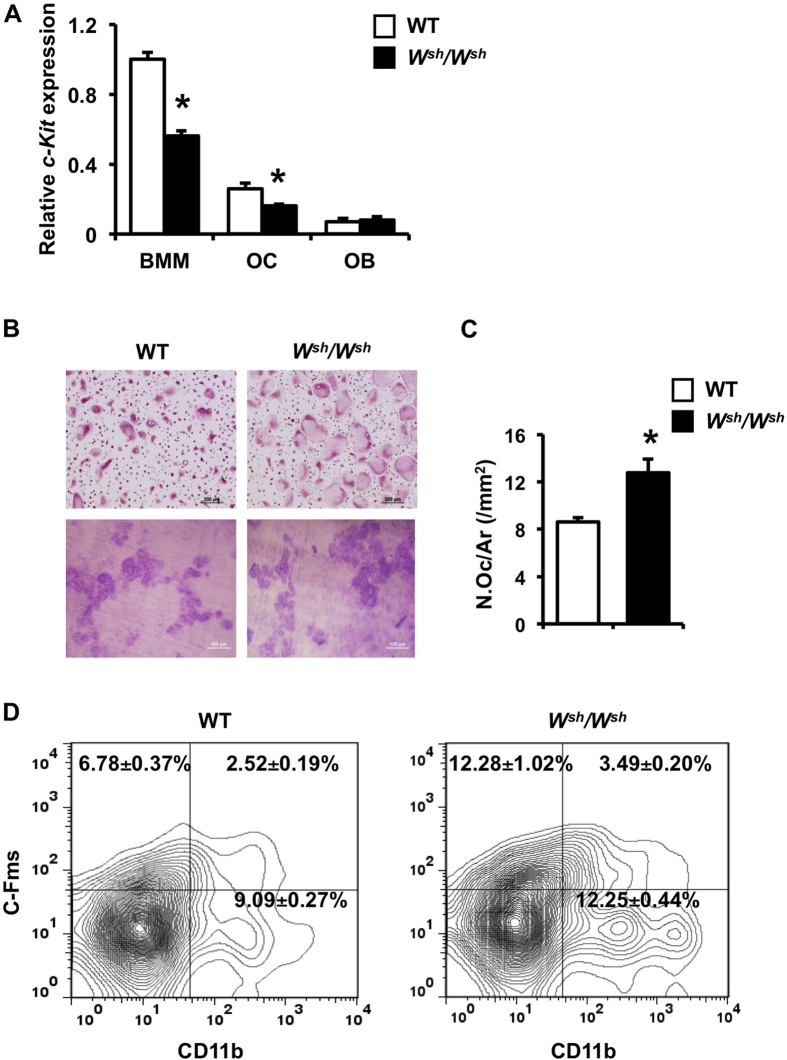
Mutation of c-*Kit* increases osteoclast precursors and differentiation but not resorbing activity. (**A**) qPCR analysis of *c-Kit* expression in BMMs, osteoclasts and osteoblasts. (**B**) Osteoclasts were generated on glass coverslips in the presence of M-CSF and RANKL and osteoclast differentiation was evaluated by TRAP staining (upper). Osteoclasts generated by co-culture with osteoblasts on collagen gel in media containing vitamin D_3_ and PGE_2_ were replated onto dentin slices and cultured for 48 h (lower). Resorption pits were stained with toluidine blue. (**C**) TRAP-positive osteoclast number per total area (/mm^2^) was quantified by OsteoMeasure software. (**D**) FACS analysis of CD11b^+^, c-Fms^+^ and CD11b^+^ cFms^+^ cells from spleen derived from WT and *W*^*sh*^*/W*^*sh*^ mice. Results are mean ± SEM. *p < 0.05 versus WT.

We examined the expression of *c-Kit* in BMM, osteoclasts, and osteoblasts. The mRNA level of *c-Kit* was much lower in osteoblasts compared with BMM and osteoclasts in *W*^*sh*^*/W*^*sh*^ mice (Fig. 4A). *c-Kit* mutation reduced the *c-Kit* mRNA levels in BMM and osteoclasts by 43 and 35%, respectively, whereas the *c-Kit* mRNA level in osteoblasts was not altered.

Mutation of *c-Kit* increased osteoclast number in all age groups. We examined whether the increased number of osteoclasts in *W*^*sh*^*/W*^*sh*^ mice was a cell-autonomous effect. Consistent with the increased *in vivo* bone resorption, TRAP staining showed increased osteoclast number in cultured BMM derived from *W*^*sh*^*/W*^*sh*^ mice compared with WT (Fig. 4B,C), indicating an intrinsic role for *c-Kit* in osteoclast differentiation. To determine the effect of *c-Kit* on osteoclast resorbing ability, equal numbers of WT and mutant osteoclasts were cultured on dentin slices and the resorbed areas were quantified. No differences were observed in resorption between the mutant and WT cells (Fig. 4B).

*W*^*sh*^*/W*^*sh*^ mice had increased osteoclast number *in vivo* and *in vitro*. To examine whether the increased osteoclast number was secondary to a greater number of osteoclast precursors, FACS analysis was performed on spleen and bone marrow cells. The results revealed that the percentages of CD11b^+^, c-Fms^+^ and CD11b^+^ c-Fms^+^ cells in *W*^*sh*^*/W*^*sh*^ spleen cells were all increased compared with WT cells (Fig. 4D). There was an increase in the CD11b^+^ bone marrow cells (23.95 ± 1.25 in WT vs 31.10 ± 1.44 in *W*^*sh*^*/W*^*sh*^ mice, *p* < 0.05), whereas c-Fms^+^ and CD11b^+^ c-Fms^+^ cells were not altered in *W*^*sh*^*/W*^*sh*^ mice.

### Mutation of *c-Kit* increases osteoblast progenitors and differentiation

**Figure 5:**
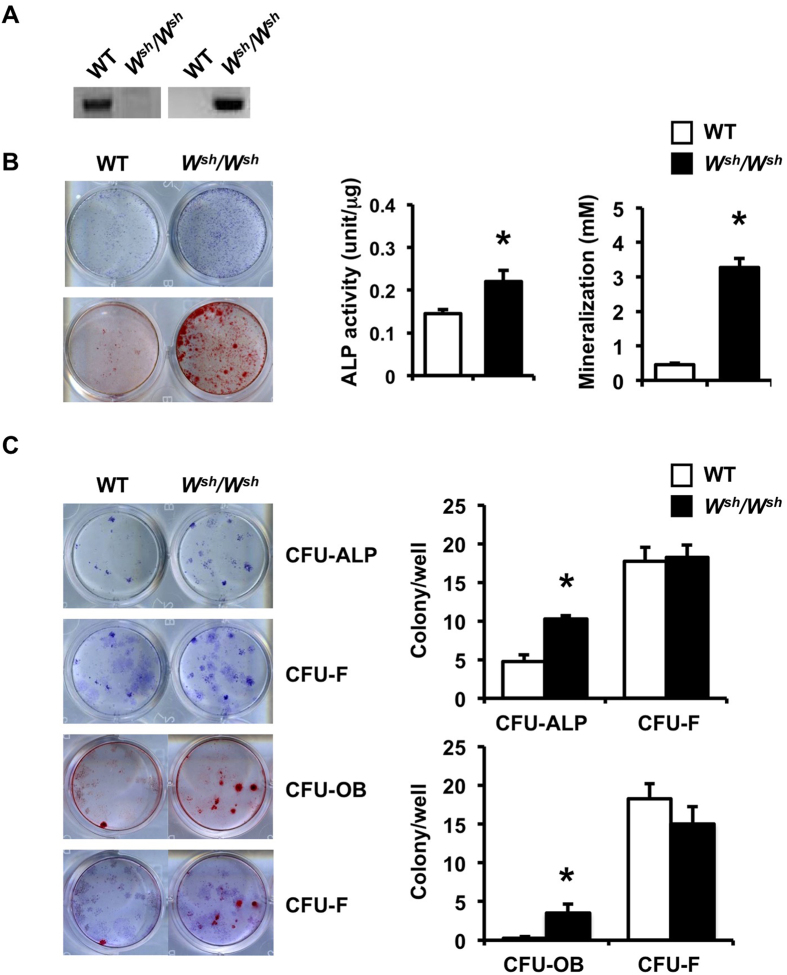
Mutation of c-*Kit* increases osteoblast differentiation and osteoprogenitors. (**A**) PCR analysis using primers to amplify product from either WT (left) or *W*^*sh*^*/W*^*sh*^ (right) genomic DNA. (**B**) ALP and alizarin red staining in calvarial osteoblasts at day 7 and 21, respectively. (**C**) The number of ALP-positive colony-forming unit (CFU-ALP) and alizarin red-positive CFU (CFU-OB) was increased in *W*^*sh*^*/W*^*sh*^ osteoblasts. The same tissue culture plates were stained with toluidine blue to determine CFU-fibroblasts (CFU-F). CFU-F number was not changed. Results are mean ± SEM. *p < 0.05 versus WT.

**Figure 6:**
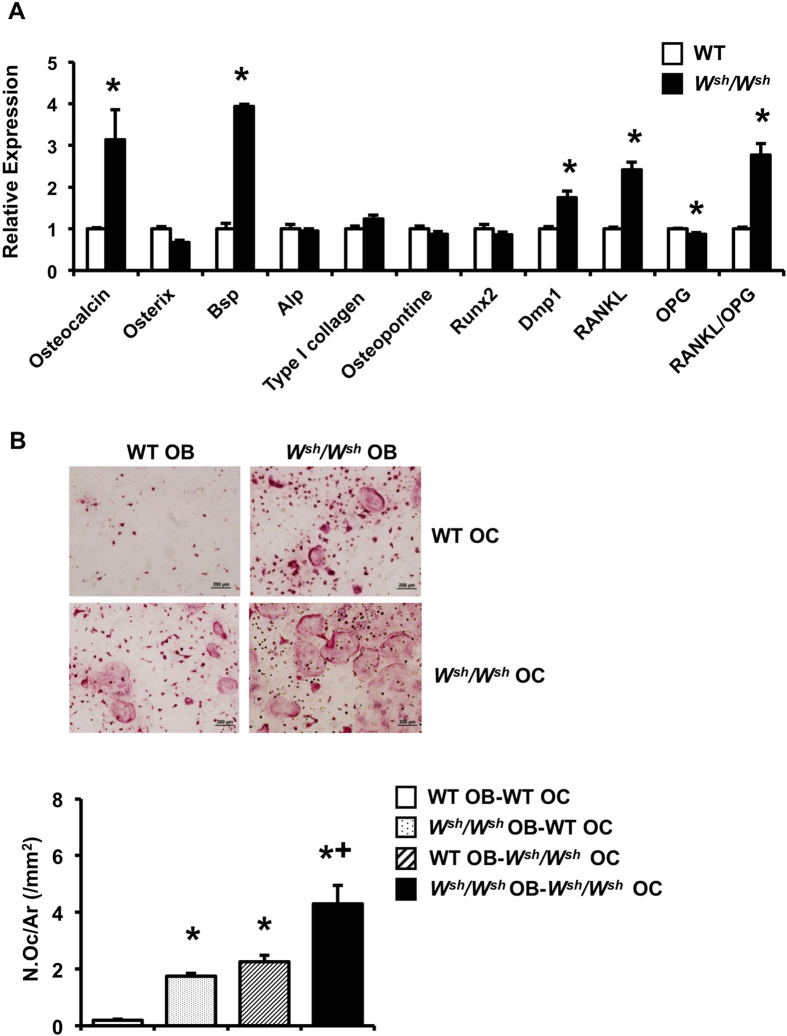
Mutation of *c-Kit* increases osteoblast marker genes *in vitro*. (**A**) qPCR analysis of mRNA expression in calvarial osteoblasts from WT and *W*^*sh*^*/W*^*sh*^ mice. (**B**) TRAP-positive osteoclast number per total area (N.Oc/Ar, /mm^2^) from co-culture of WT or *W*^*sh*^*/W*^*sh*^ osteoblasts with WT or *W*^*sh*^*/W*^*sh*^ BMMs. OB; osteoblast and OC; osteoclast. Results are mean ± SEM. *p < 0.05 versus WT, and +p < 0.05 versus *W*^*sh*^/*W*^*sh*^ OB-WT OC and WT OB-*W*^*sh*^/*W*^*sh*^ OC.

To determine whether the increased bone formation observed in growing *W*^*sh*^*/W*^*sh*^ mice was due to enhanced osteoblast proliferation and/or differentiation, we analyzed the proliferation and differentiation of calvarial osteoblasts *in vitro*. One-day-old pups were genotyped (Fig. 5A). Osteoblasts derived from *W*^*sh*^*/W*^*sh*^ mice had higher ALP activity and formed more mineralized bone nodules than those from WT controls (Fig. 5B), whereas osteoblast proliferation, determined by BrdU labeling, was unchanged (data not shown). Because *c-Kit* expression was similar in *W*^*sh*^*/W*^*sh*^ and WT osteoblasts, we hypothesized that an increase in the osteoblast precursor population contributed to increased osteoblast differentiation in *W*^*sh*^*/W*^*sh*^ mice. To test this hypothesis, we analyzed the capability of calvarial osteoblasts derived from *W*^*sh*^*/W*^*sh*^ mice and their WT littermates to form ALP-positive colony forming units (CFU-ALP) and CFU-osteoblasts (CFU-OB). We found that although CFU-ALP and CFU-OB were increased, the number of CFU-fibroblasts (CFU-F) was not changed in *W*^*sh*^*/W*^*sh*^ osteoblasts compared with WT controls (Fig. 5C). qPCR analysis of calvarial osteoblasts derived from *W*^*sh*^*/W*^*sh*^ mice showed increased expression of the osteoblast marker genes osteocalcin, Bsp, and Dmp1 (Fig. 6A). The mRNA level for RANKL increased, whereas OPG mRNA level decreased, leading to an increase in the RANKL/OPG mRNA ratio in *W*^*sh*^*/W*^*sh*^ osteoblasts. To further investigate whether the increased RANKL/OPG ratio in these cells enhanced osteoclast differentiation, co-culture of *W*^*sh*^*/W*^*sh*^ or WT osteoblasts with *W*^*sh*^*/W*^*sh*^ or WT BMMs was performed (Fig. 6B). Compared with WT osteoblasts, *W*^*sh*^*/W*^*sh*^ osteoblasts induced greater osteoclast differentiation regardless of the BMM genotype, suggesting that the increased RANKL/OPG ratio in *W*^*sh*^*/W*^*sh*^ osteoblasts led to increased osteoclast differentiation.

### Osteoclast-coupling factor Wnt 10b is increased in *W* ^*sh*^ */W* ^*sh*^osteoclasts

**Figure 7:**
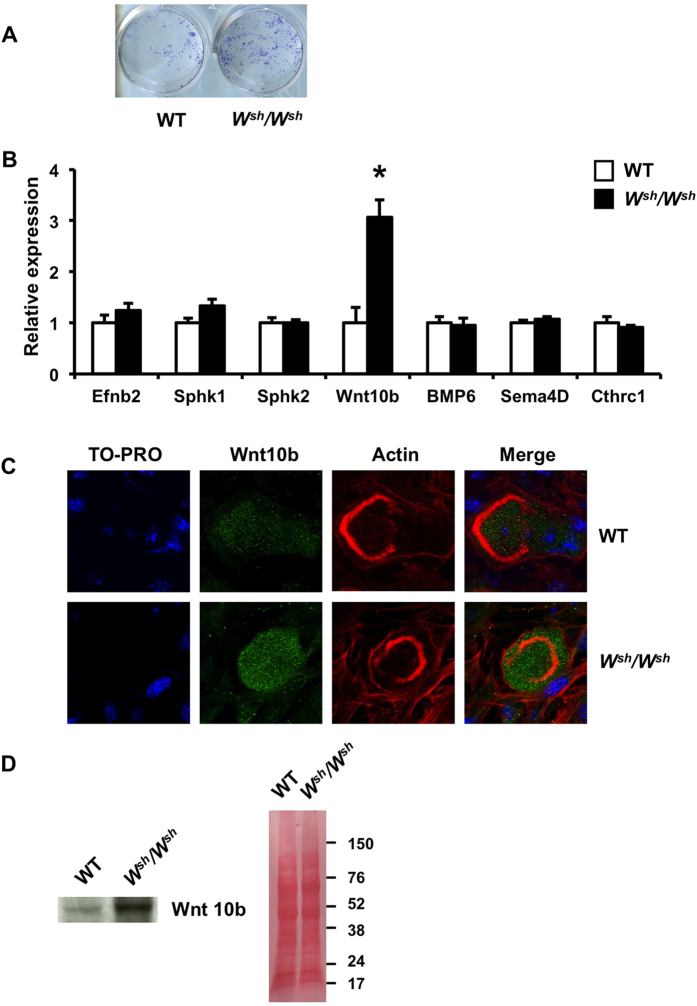
Mutation of *c-Kit* increases Wnt10b mRNA expression and protein level in osteoclasts. (**A**) ALP staining of osteoblast cultures treated with conditioned medium derived from either *W*^*sh*^*/W*^*sh*^ or WT osteoclasts (**B**) qPCR analysis of osteoclast mRNA expression. (**C**) Confocal immunofluorescence images of osteoclasts generated on dentin slices and immunolabeled for nuclei (TO-PRO3, blue), Wnt10b (green) and actin (rhodamine phalloidin, red). (**D**) Western blot analysis of conditioned medium from WT and *W*^*sh*^*/W*^*sh*^ osteoclast was performed with an anti-Wnt10b antibody (left). The same membrane was stained with Ponceau S (right) as a loading control. Results are mean ± SEM. *p < 0.05 versus WT.

We examined whether altered local regulation between osteoblasts and osteoclasts contributed to increased osteoanabolic factors and subsequent bone formation in *W*^*sh*^*/W*^*sh*^ mice. WT calvarial osteoblasts were cultured in conditioned medium from either *W*^*sh*^*/W*^*sh*^ or control osteoclasts. Calvarial osteoblasts treated with conditioned medium derived from *W*^*sh*^*/W*^*sh*^ osteoclast cultures demonstrated increased ALP staining, indicating that the conditioned medium contained more anabolic coupling factors (Fig. 7A). We then determined the expression of the known osteoclast-derived coupling factor genes, Efnb2, Sphk1, Sphk2, BMP6, Sema4D, Cthrc1 and Wnt10b^21^,^22^,^23^,^24^,^25^,^26^ from *W*^*sh*^*/W*^*sh*^ and WT osteoclasts using qPCR. As shown in Fig. 7B, *c-Kit* mutants increased their expression of Wnt10b but not that of Efnb2, Sphk1, Sphk2, BMP6, Sema4D, or Cthrc1. Confocal immunofluorescence analysis of osteoclasts revealed increased Wnt10b staining in *W*^*sh*^*/W*^*sh*^ osteoclasts cultured on dentin slices (Fig. 7C). Western blot analysis of osteoclast-conditioned media confirmed an increase in Wnt10b protein level in mutants (Fig. 7D). Before blotting, the membranes were stained with Ponceau S for protein detection. We found that the protein levels were comparable between the *W*^*sh*^*/W*^*sh*^ and WT conditioned medium.

### Blocking Wnt10b signaling decreased *W* ^*sh*^ */W* ^*sh*^osteoclast conditioned medium-induced ALP activity and mineralization

**Figure 8:**
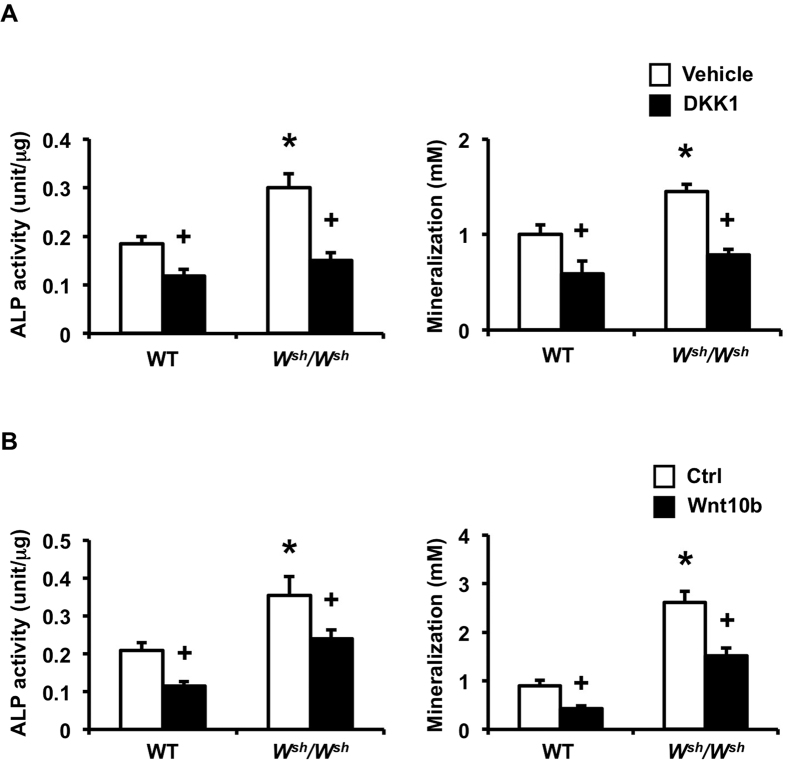
Blocking Wnt10b attenuates ALP activity and mineralization induced by *W*^*sh*^*/W*^*sh*^ osteoclast-conditioned medium. (**A**) Calvarial osteoblasts were treated with either WT or *W*^*sh*^*/W*^*sh*^osteoclast-conditioned medium with or without DKK1. ALP activity (*n* = 5 per group) and mineralization (*n* = 5 per group) were quantified. (**B**) Osteoblasts were cultured with either WT or *W*^*sh*^*/W*^*sh*^osteoclast-conditioned medium pretreated with either isotype control (Ctrl) or Wnt10b antibody (Wnt10b). ALP activity (*n* = 5 per group) and mineralization (*n* = 5 per group) were quantified. Results are mean ± SEM. *p < 0.05 versus WT and ^+^ *p* < 0.05 versus corresponding controls.

To evaluate whether osteoclast-derived Wnt10b contributed to the coupling-mediated enhancement of osteoblast formation, 0.2 μg/ml DKK1, a Wnt antagonist, was added to the WT calvarial osteoblast cultures with conditioned medium from either *W*^*sh*^*/W*^*sh*^ or control osteoclasts. DKK1 markedly inhibited the increase in ALP activity and mineralization induced by *W*^*sh*^*/W*^*sh*^ osteoclast conditioned medium (Fig. 8A). To further confirm the effects of the Wnt antagonist, we used antibody to neutralize the influence of Wnt10b on ALP activity and mineralization. Neutralizing Wnt10b inhibited the *W*^*sh*^*/W*^*sh*^ osteoclast conditioned medium-induced increase in ALP activity and mineralization. Therefore, Wnt10b is the major coupling factor responsible for *W*^*sh*^*/W*^*sh*^ osteoclast-mediated increase in bone formation.

## Discussion

Our data suggest that c-Kit plays a crucial role in bone remodeling process. In the present study, we first examined the skeletal phenotype of *W/W*^*v*^ mice that carry a *c-Kit* point mutation and are sterile. *W/W*^*v*^ mutants had decreased cortical and cancellous bone volume. The reduction in cancellous bone volume was the result of a marked decrease in osteoblast surface and increase in osteoclast surface, indicating uncoupled bone turnover. To gain further insight into the precise role of *c-Kit* in bone metabolism, we used *W*^*sh*^*/W*^*sh*^ mice that possess an inversion mutation upstream of the *c-Kit* region and are fertile. This *c-Kit* mutation, which reduced *c-Kit* expression in BMMs and osteoclasts but did not affect its expression in osteoblasts, resulted in osteopenia associated with increased bone formation and increased bone resorption in growing *W*^*sh*^*/W*^*sh*^ mice. The skeletal phenotype was milder when animals were mature. The increased osteoclast number was a consequence of an increased RANKL/OPG ratio in osteoblasts. It appears that the alteration in the osteoclast-osteoblast coupling mechanism contributes to increased bone formation in *W*^*sh*^*/W*^*sh*^ mice. Mutation of *c-Kit* stimulates Wnt10b secretion from osteoclasts that promotes osteoblast mineralization and subsequently bone formation. Blocking Wnt10b markedly inhibited the increased ALP activity and mineralization that were induced by *W*^*sh*^*/W*^*sh*^osteoclast conditioned medium.

Physiologically, *c-Kit* expression is tightly regulated and the reduction or loss of *c-Kit* activity by known mutations is associated with bone loss. Osteoclasts express c-*Kit* receptor on their cell membrane and respond to its ligand directly through cell-to-cell contact^7^ or indirectly through paracrine factors. The skeletal phenotype of *W/W*^*v*^ mice is subtle and these mice are infertile due to germ cell depletion. It has been reported that male *W/W*^*v*^ mice have normal plasma testosterone levels but elevated FSH levels^27^. However, our data indicated that seminal vesicle weight, an index of androgen deficiency, decreased by 42% in *W/W*^*v*^ mice. The serum testosterone was also decreased in these mice. Male hypogonadism increases the production of osteoclasts and osteoblasts, leading to an increase in cancellous bone turnover^28^,^29^,^30^. These changes were quite distinct from those found in *W/W*^*v*^ mice. Thus, the low bone mass observed in growing *W/W*^*v*^ mice is unlikely to be solely the consequence of androgen deficiency. *W*^*sh*^*/W*^*sh*^ mice that are fully fertile and have normal testosterone level also exhibited osteopenia. However, the cellular mechanism of bone loss in these mice was different from that of *W/W*^*v*^ mice. Increased osteoclast number and increased osteoblast function with a net increase in bone resorption contributed to the skeletal phenotype observed in *W*^*sh*^*/W*^*sh*^ mice.

Bone undergoes renewal and repair through bone remodeling process, with no net change in bone volume when the amount of bone removed is precisely replaced by that of bone formed. Failure to elicit a corresponding increase in bone formation following a dramatic increase in osteoclasts causes net bone loss in growing male *W*^*sh*^*/W*^*sh*^ mice. *c-Kit* mutation decreased the mRNA level of *c-Kit* in BMMs and osteoclasts leading to increased osteoclast differentiation *in vitro*. These results suggest a cell-autonomous effect in *W*^*sh*^*/W*^*sh*^ mice. Osteoclast formation is driven by the key effector RANKL derived from osteoblasts or other cell lineages within the bone microenvironment. RANKL activity is moderated by a decoy receptor OPG. *c-Kit* mutation induced bone resorption by increasing RANKL expression in both *in vivo* and *in vitro*. *W*^*sh*^*/W*^*sh*^ osteoblasts had an increased RANKL/OPG mRNA ratio, which has been shown to promote osteoclastogenesis^31^,^32^. Our co-culture experiments using osteoblasts and osteoclasts also confirmed that the increased RANKL/OPG ratio in *W*^*sh*^*/W*^*sh*^ osteoblasts was responsible for the increased osteoclast differentiation observed *in vitro*.

According to the osteoclast commitment and differentiation pathway, CD11b is expressed during the differentiation of mononuclear early progenitor cells to mature multinucleated osteoclasts. The expression is higher in mononuclear cells and low in mature osteoclasts. It has been reported that c-Fms is a major determinant in osteoclast differentiation^33^,^34^. FACS analysis of spleen cells derived from *W*^*sh*^*/W*^*sh*^ mice revealed an increase in the percentage of CD11b^+^, c-Fms^+^, and CD11b^+^ c-Fms^+^ cells. Although the number of c-Fms^+^, and CD11b^+^ c-Fms^+^ cells in *W*^*sh*^*/W*^*sh*^ bone marrow was not altered, CD11b^+^ cell number was increased. These data suggest that reduced c-Kit signaling acts to expand the pool of osteoclast precursors, leading to increased osteoclast differentiation and bone resorption in *W*^*sh*^*/W*^*sh*^ mice. Although histomorphometric analysis indicated no changes in bone architecture at 9 weeks of age, three-dimensional μCT showed decreases in cancellous bone volume, trabecular thickness and trabecular number with concomitant increase in trabecular separation. The CTX level in *W*^*sh*^*/W*^*sh*^ mice was increased by 68, 44 and 41% at 6, 9 and 13 weeks old, respectively, indicating a reduction in osteoclast activity in the mutants as the animals grew.

The mechanism by which loss-of-function mutation of *c-Kit* led to osteopenia in *W*^*sh*^*/W*^*sh*^mice remains unclear. It has been reported that c-Kit mediates cell-to-cell interactions between osteoclasts and osteoblasts/stromal cells through membrane bound KL^7^. Soluble KL, in concert with other factors, stimulates osteoclast formation and activity^35^. Gleevec, which inhibits c-Kit as well as c-Fms, c-Abl, and PDGF receptor^36^,^37^, decreases osteoclast number in rodents^38^ and inhibit osteoclast differentiation *in vitro*^39^. When used therapeutically to treat chronic myeloid leukemia, it sometimes induces secondary hyperparathyroidism with inconsistent reports of changes in bone formation and bone resorption^40^,^41^. The extent to which the effects of Gleevec on bone are mediated by inhibition of c-Kit has not been determined.

*W/W*^*v*^ and *W*^*sh*^*/W*^*sh*^ mice have reduced numbers of mast cells in various soft tissues^42^,^43^,^44^. Mast cell deficiency is associated with low bone turnover, whereas excessive mast cell number induces bone loss^45^. Although the relationship between mast cells and osteoclasts is not completely understood, mast cells can modulate osteoclast activity through their release of granule-associated cytokines. However, it has been reported that mast cells are rarely found in mouse bone marrow^18^,^46^. Therefore, it is unlikely that mast cells contribute to the skeletal changes observed in our study.

The role of *c-Kit* in bone formation remains undefined. Although it has been reported that *c-Kit* is expressed in primary rat osteoblasts and SaOS-2 cells but not MC3T3-E1, ST2 or RAW 264.7 cells^47^, we found that *c-Kit* expression was much lower in mouse calvarial osteoblasts compared with osteoclasts. The *W*^*sh*^ mutation affects the tissue-specific expression of *c-Kit* during embryonic development and adulthood^16^. Although *c-Kit* expression in *W*^*sh*^*/W*^*sh*^ osteoclasts was decreased by 35%, its expression in osteoblasts was not altered, indicating that the increased bone formation in *W*^*sh*^*/W*^*sh*^ mice was not due to an intrinsic effect in osteoblasts. The fact that calvarial osteoblasts isolated from *W*^*sh*^*/W*^*sh*^ mice had increased ALP activity and formed more bone nodules together with an increase in the number of CFU-ALP and CFU-OB without any change in total CFU-F suggests that the increased osteoblast number in bone *in vivo* resulted from an increase in the number of committed osteoblast precursors. Our finding indicates that *c-Kit* mutation leads to increased bone formation in *W*^*sh*^*/W*^*sh*^ mice through indirect osteoclast-mediated effects. The evidence that bone resorption triggers bone formation suggests that certain coupling factors derived from osteoclasts are responsible for recruiting osteoblasts progenitors to the remodeling site and stimulating bone formation. Our finding that conditioned medium derived from *W*^*sh*^*/W*^*sh*^ osteoclast culture increased ALP activity and mineralization in osteoblasts confirmed an increase in osteoclast-secreted osteoanabolic factors. We demonstrated that *c-Kit* mutation stimulated Wnt10b, a known coupling factor, production, and secretion from osteoclasts. The increase in Wnt10b production in *c-Kit* mutant osteoclasts, together with the increased osteoblast differentiation induced by *W*^*sh*^*/W*^*sh*^ osteoclast-conditioned medium and the increased bone formation *in vivo*, strengthen the evidence that osteoclasts can enhance bone formation through secreted coupling factors. Binding of Wnt10b to Wnt receptors, LRP-5 and LRP-6, on osteoblasts stimulates new bone formation^48^. Antagonizing Wnt10b blunted the anabolic effects of the osteoclast-conditioned medium *in vitro*. Therefore, it is likely that Wnt10b is an osteoclast-derived molecule responsible for the enhancement of bone formation in *W*^*sh*^*/W*^*sh*^ mice. However, the mechanism by which *c-Kit* mutation regulates Wnt10b production by osteoclasts remains to be determined. Our findings do not exclude a contribution of matrix-derived growth factors, such as TGF-β1, released from the bone matrix during bone resorption. Other investigators have shown that TGF-β1 stimulates Wnt10b production in osteoclasts that enhances the coupling of bone resorption with formation^26^. Further studies are required to address this question.

In conclusion, this study is the first to report the importance of *c-Kit* as a negative regulator of bone turnover and that Wnt10b is a physiologically important osteoclast-secreted molecule that promotes bone formation in *c-Kit* mutants. Targeting *c-Kit* may provide a new insight to develop therapeutic intervention for skeletal disorders.

## Materials and Methods

### Animals

*W*^*sh*^/*W*^*sh*^, *W/W*^*v*^ and WBB6F1/J-*Kit*^+/+^ wildtype (WT) mice were purchased from Jackson Laboratory (Bar Harbor, ME, USA). *W*^*sh*^/*W*^*sh*^ mice were crossed to C57BL6/J (Jackson Laboratory) to produce heterozygotes. *W*^*sh*^/ ^+^ mice were then crossed to generate *W*^*sh*^/*W*^*sh*^ mice and littermate controls. *W/W*^*v*^ and *W*^*sh*^/*W*^*sh*^ mice are white, and black-eyed, whereas their controls are black. Male and female mice were fed standard mouse chow *ad libitum* and maintained under a 12:12 h light/dark cycle. Animals were maintained in accordance with the Guide for the Care and Use of Laboratory Animals of the National Institutes of Health. The experimental protocols were approved by the Institutional Animal Care and Use Committee at the Harvard Medical School.

Mice were subcutaneously injected with 20 mg/kg calcein (Sigma, St Louis, MO, USA) and 40 mg/kg demeclocycline (Sigma) and the interlabeling periods were 4, 5, and 6 days for 6-, 9-, and 13-week-old mice, respectively. At the end of the experiment, the mice were weighed and anesthetized with isoflurane. Blood samples were collected and centrifuged and the serum was kept at −80 °C for determination of P1NP and CTX. The seminal vesicles, tibiae, and femora were removed. The right femora and tibiae of *W/W*^*v*^ mice were fixed in 70% alcohol for μCT analysis and bone histomorphometry, respectively. For *W*^*sh*^/*W*^*sh*^ mice, the left tibiae were used for μCT analysis, whereas the right tibiae were analyzed for bone histomorphometry. The left femora of *W*^*sh*^*/W*^*sh*^ mice were frozen in liquid nitrogen and stored at −80 °C until processed for RNA isolation and qPCR analysis.

### Histomorphometry

The proximal metaphyses of the right tibiae were dehydrated in acetone, infiltrated, and embedded without demineralization in methyl methacrylate. Undecalcified longitudinal 5 μm thick sections were cut on a Reichert-Jung Supercut 2165 microtome (Leica) and mounted unstained for dynamic measurements. Mineralizing surface per bone surface (MS/BS, %) and mineral apposition rate (MAR) were measured. Bone formation rate (BFR) was calculated as the product of MS/BS and MAR and expressed per bone surface (BFR/BS, (μm^3^/μm^2^/year), bone volume (BFR/BV, %/year) and tissue volume referent (BFR/TV, %/year). Consecutive sections were toluidine blue stained to quantify osteoblast number and osteoclast number. Bone volume per tissue volume (BV/TV, %), trabecular thickness (Tb.Th, μm), trabecular separation (Tb.Sp, μm), and trabecular number (Tb.N, /mm) were measured. Histomorphometric measurements were carried out using the OsteoMeasure system (OsteoMetric Inc.) and all parameters were expressed according to standardized nomenclature^49^.

### μCT

A three-dimensional reconstruction of bone microarchitecture was performed using a desktop μCT35 (Scanco Medical) following the recommended guidelines^50^. Cortical bone at the tibial or femoral midshaft and cancellous bone at the proximal tibial metaphysis or femoral distal metaphysis were scanned using a 7-μm isotropic voxel size, 50 kVp, and 144 μA. For cortical bone, 86 transverse μCT slices were evaluated using a threshold at 35% of the maximal gray scale value to assess the total cross-sectional volume (mm^3^), cortical volume (mm^3^), marrow volume (mm^3^), and cortical thickness (mm). Cancellous bone was assessed in 464 transverse slices using a fixed threshold at 29% of the maximal gray scale value. Measurements included bone volume fraction (BV/TV, −), trabecular number (Tb.N,/mm), trabecular thickness (Tb.Th, mm) and trabecular separation (Tb.Sp, mm), connectivity density (ConnD,/mm^3^), and structural model index (SMI, −).

### Osteoblast differentiation and proliferation and CFU assays

One-day old pups were genotyped by PCR using primer pair P1 (TTTGCACGTGCTAGTTACAC) and P2 (TTAAGATGGCACCCTGCTG) for WT template and primer pair P3 (AGGCTTGCAGCGCATTAT) and P4 (GAGGATTCATAGTTGTTCAATGTCC) for *W*^*sh*^/*W*^*sh*^ template^51^. Primary calvarial osteoblasts derived from *W*^*sh*^/*W*^*sh*^ mice and their control littermates were plated at 1 × 10^4^ cells/well in 12-well plates and cultured in differentiation medium containing α-MEM supplemented with 10% FBS, 100 unit/ml penicillin and 100 μg/ml streptomycin, 5 mM β-glycerophosphate, 10 μM dexamethasone, and 50 μg/ml ascorbic acid. The cells were fixed with 3.7% formaldehyde, and stained for ALP and bone nodules with Fast Blue Alkaline phosphatase (Sigma) and 2% alizarin red (Sigma) on days 7 and 21, respectively. ALP activity and mineralized bone nodules were quantified as previously described^52^. For the DKK1 experiments, WT calvarial osteoblasts were plated at 0.5 × 10^4^ cells/well in 24-well plates and cultured with osteoclast-conditioned medium. Recombinant DKK1 (R&D systems) at 0.2 μg/ml was added to the medium and ALP activity and mineralization bone nodules were determined. For the Wnt10b neutralization experiments, osteoclast-conditioned medium was pretreated with either isotype control or Wnt10b antibody (R&D) at 4 μg/ml before addition to the osteoblast cultures. The cells were stained with ALP and alizarin red.

Calvarial osteoblasts derived from *W*^*sh*^/*W*^*sh*^ mice and controls (7 × 10^3^ cells/well) were cultured in 96-well plates for 24 h and exposed to BrdU labeling reagent for 18 h. Osteoblast proliferation was determined by a BrdU incorporation assay per the manufacturer’s instructions (Cell Signaling). For CFU assays, calvarial osteoblasts derived from *W*^*sh*^/*W*^*sh*^and WT littermates were plated at 0.25 × 10^4^ cells/well in 12-well plates and cultured in differentiation medium. CFU-ALP was determined as ALP-positive colonies on day 7. Mineralized nodules were stained with alizarin red on day 23 for the determination of CFU-OB. The cells were then stained with toluidine blue to determine CFU-F on days 7 and 23 in the same tissue culture plate.

### Preparation of osteoclasts

Bone marrow cells were cultured in α-MEM containing 10%FBS, 100 unit/ml penicillin and 100 μg/ml streptomycin for 24 h to generate BMMs. The BMMs were then cultured on tissue culture plastic or coverslips in α-MEM for 2 days with 20 ng/ml M-CSF and for an additional 6 days in the same medium with 20 ng/ml M-CSF and 3.3 ng/ml RANKL. Osteoclast-conditioned medium was collected. Multinucleated cells were identified by TRAP staining. For the resorption assay, osteoclasts were generated by culturing BMMs with primary CD1 calvarial osteoblasts in media containing 10 nM 1,25-dihydroxyvitamin D_3_ and 1 μM prostaglandin E_2_ on a collagen gel. The collagen was digested with 0.1% collagenase and the osteoclasts were replated onto dentin slices for an additional 48 h to determine their bone-resorbing activity using toluidine blue staining.

For the co-culture studies, calvarial osteoblasts derived from WT and mutants were cultured in α-MEM containing 10% FBS, 100 unit/ml penicillin and 100 μg/ml streptomycin for 24 h. BMMs were added and cultured in media containing 1,25-dihydroxyvitamin D_3_ and prostaglandin E_2_ for 5 additional days. TRAP staining was performed.

### qPCR

Total RNA was isolated from femora using Trizol reagent according to the manufacturer’s instructions (Invitrogen) and purified using an RNeasy Mini kit (Qiagen). The RNA yields were determined spectrophotometrically at 260 nm. One μg of total RNA was used to synthesize cDNA using SuperScript VILO (Invitrogen). The qPCR was performed at 57 °C for 40 cycles using an iCycler (Biorad) and the results were normalized to GAPDH expression. The primer sequences used are shown in Supplementary Table S5.

### FACS analysis

Bone marrow and spleen cells were removed and red blood cells were lysed with lysis buffer (eBioscience). The cells were incubated with Alexa Fluor® 488-conjugated anti-mouse CD11b (eBioscience) for 30 minutes at 4 °C and washed twice with washing buffer. The cells were incubated with PE-conjugated anti-mouse c-Fms (eBioscience) for 30 minutes and washed twice. The stained cells were suspended in PBS and flow cytometry was performed using BD LSRFortessa (Becton Dickinson).

### Confocal microscopy

Osteoclasts plated on dentin slices were fixed with 3.7% formaldehyde in phosphate-buffered saline (PBS) for 10 min. Cells were permeabilized in PBS containing 0.05% saponin, 0.1% BSA and 5% normal serum for 30 min, incubated with anti-mouse/rat/human Wnt10b antibody (Santa Cruz) for 1 h, washed, incubated with fluorescent secondary antibody (Alexa Fluor 488), washed again, and mounted in FluorSave (Calbiochem). For actin labeling, the cells were incubated in a 1:40 dilution of rhodamine phalloidin stock solution (Invitrogen) for 1 h and washed with PBS. The nuclei were labeled with TO-PRO-3 (1:1000) in the rhodamine phalloidin solution. Osteoclasts were visualized using a 510 Meta laser scanning confocal microscope (Carl Zeiss) and images were recorded.

### Western blot analysis

Osteoclasts were generated in a 6-well-plate and conditioned medium was collected and concentrated 50-fold using Amicon ultra centrifuge filter units (Millipore). Protein concentration was determined using a BCA protein assay kit (Thermo Scientific). Samples (200 μg) were resolved using 4–12% Mini-PROTEAN TGX precast gel (Biorad) and transferred to nitrocellulose membranes. The membranes were stained with Ponceau S dye for protein detection then washed in TBST buffer (50 mM Tris-HCl, pH 7.5, 150 mM NaCl, and 0.1% Tween 20) and incubated in TBST buffer containing anti-Wnt10b antibody overnight at 4 °C. The membranes were washed in TBST and incubated with anti–rabbit IgG horseradish peroxidase–conjugated secondary antibody (Promega). The membranes were then washed and developed with an enhanced chemiluminescence detection kit (PerkinElmer, Inc.).

### Serum analysis

Serum P1NP and CTX were determined using Rat/Mouse P1NP and RatLaps^TM^ EIA kit, respectively per the manufacturer’s protocol (Immunodiagnostic systems). Serum testosterone concentration was measured by enzyme-linked immunosorbent assay (ELISA) per the manufacturer’s directions (R&D Systems).

### Statistical analysis

All data are expressed as the mean ± SE. Unpaired Student’s *t*-test was used to compare between 2 groups. Multiple comparisons were determined using one-way ANOVA followed by Fisher’s protected least significant difference test. The *in vitro* experiments were repeated three times. Statistical significance was considered at *p* < 0.05.

## Additional Information

**How to cite this article**: Lotinun, S. and Krishnamra, N. Disruption of c-*Kit* Signaling in *Kit^W-sh/W-sh^*  Growing Mice Increases Bone Turnover. *Sci. Rep.* **6**, 31515; doi: 10.1038/srep31515 (2016).

## Supplementary Material

Supplementary Information
