# Supplementary material for: Disruption of c-Kit Signaling in KitW-sh/W-sh Growing Mice Increases Bone Turnover
Source: Sci Rep. 2016 Aug 16;6:31515. doi: 10.1038/srep31515 (PMC4985756; doi:10.1038/srep31515)
Supplement: Supplementary Information [file srep31515-s1.pdf]

Disruption of c-*Kit* Signaling in *Kit*$^{W\text{-}sh/W\text{-}sh}$ Growing Mice Increases Bone Turnover

Sutada Lotinun[1,2]*& Nateetip Krishnamra[3]

[1]Department of Physiology and STAR on Craniofacial and Skeletal Disorders, Faculty of Dentistry, Chulalongkorn University, Bangkok, Thailand
[2]Department of Oral Medicine, Infection and Immunity, Harvard School of Dental Medicine, Boston, MA, USA
[3]Department of Physiology, Faculty of Science, Mahidol University, Bangkok, Thailand

*Address correspondence to:

Sutada Lotinun, Ph.D.
Department of Physiology
Faculty of Dentistry
Chulalongkorn University
Bangkok, Thailand
Email: sutada.l@chula.ac.th

Table S1. μCT analysis of 6-wk-old male *W/W^v* mice and their control littermates

Parameters	WT (n=7)	*W/W^v* (n=7)
Cortical Bone (midshaft femur)		
Total cross-sectional volume (mm^3)	1.16±0.03	0.92±0.02*
Cortical volume (mm^3)	0.49±0.01	0.38±0.01*
Marrow volume (mm^3)	0.42±0.00	0.41±0.01
Cortical thickness (mm)	0.173±0.004	0.148±0.004*
Cancellous Bone (distal femur)		
BV/TV (-)	0.21±0.01	0.11±0.01*
Tb.Th (mm)	0.039±0.000	0.035±0.002*
Tb.N (/mm)	6.35±0.13	3.69±0.29*
Tb.Sp (mm)	0.16±0.00	0.29±0.03*
Conn.D (/mm^3)	613±38	398±24*
SMI (-)	1.19±0.11	1.80±0.08*

Results are mean ± SEM
*p<0.05 compared with WT, unpaired *t* test

Table S2. μCT analysis of 6-, 9- and 13-wk-old male W^{sh}/W^{sh} mice and their control littermates

Parameters	6-week-old		9-week-old		13-week-old	
	WT (n=6)	W^{sh}/W^{sh} (n=7)	WT (n=8)	W^{sh}/W^{sh} (n=7)	WT (n=4)	W^{sh}/W^{sh} (n=7)
Cortical Bone (midshaft tibia)						
Total cross-sectional volume (mm^3)	0.52±0.02	0.44±0.01*	0.70±0.02	0.70±0.01	0.69±0.06	0.70±0.03
Cortical volume (mm^3)	0.27±0.01	0.24±0.01*	0.42±0.01	0.42±0.01	0.42±0.03	0.42±0.02
Marrow volume (mm^3)	0.24±0.01	0.21±0.01*	0.28±0.01	0.28±0.01	0.27±0.03	0.27±0.01
Cortical thickness (mm)	0.161±0.003	0.151±0.004	0.221±0.003	0.215±0.004	0.221±0.005	0.227±0.006
Cancellous Bone (proximal tibia)						
BV/TV (-)	0.11±0.01	0.08±0.01*	0.18±0.01	0.14±0.01*	0.14±0.01	0.14±0.02
Tb.Th (mm)	0.032±0.001	0.029±0.001	0.044±0.001	0.038±0.001*	0.040±0.001	0.043±0.001
Tb.N (/mm)	4.53±0.14	3.59±0.07*	5.77±0.06	5.19±0.12*	5.07±0.08	4.62±0.36
Tb.Sp (mm)	0.22±0.01	0.28±0.01*	0.16±0.00	0.18±0.01*	0.19±0.00	0.22±0.03
Conn.D (/mm^3)	297±31	214±19*	295±8	256±13*	187±14	173±20
SMI (-)	2.14±0.12	2.30±0.08	1.83±0.07	1.87±0.05	1.91±0.19	1.67±0.08

Results are mean ± SEM

*p<0.05 compared with corresponding WT, unpaired *t* test

Table S3. Histomorphometric analysis of tibiae from 6-, 9- and 13-week-old male W^{sh}/W^{sh} mice and their control littermates

Parameters	6-week-old		9-week-old		13-week-old	
	WT (n=7)	W^{sh}/W^{sh} (n=7)	WT (n=7)	W^{sh}/W^{sh} (n=7)	WT (n=7)	W^{sh}/W^{sh} (n=7)
BV/TV (%)	9.76±0.77	6.68±1.13*	9.72±0.84	10.31±0.74	9.99±1.30	10.74±0.63
Tb.Th (μm)	32.47±1.07	30.77±1.79	32.59±2.20	34.30±1.43	33.96±2.15	35.50±1.23
Tb.N (/mm)	3.00±0.20	2.16±0.33*	2.98±0.15	3.00±0.15	2.89±0.24	3.02±0.12
Tb.Sp (μm)	312±28	494±81*	308±17	304±17	325±29	299±14
MS/BS (%)	25.53±1.01	29.14±2.09	35.24±1.96	36.67±1.78	28.73±3.47	31.78±2.99
MAR (μm/day)	2.11±0.07	2.41±0.08*	1.54±0.08	2.07±0.08*	1.34±0.11	1.46±0.06
BFR/BS ($\mu m^3/\mu m^2$/year)	196±11	256±19*	197±11	278±20*	136±13	173±23
BFR/BV (%/year)	1129±79	1552±115*	1069±71	1570±83*	786±73	979±115
BFR/TV (%/year)	115±12	108±17	124±8	165±10*	79±7	96±12
Ob.S/BS (%)	8.73±0.72	16.06±2.85*	11.13±1.10	9.21±1.12	3.05±0.54	3.30±0.60
N.Ob/T.Ar (/mm^2)	38.03±2.41	53.90±10.19	52.33±6.87	40.35±4.15	14.36±2.15	15.97±3.16
N.Ob/B.Pm (/mm)	6.54±0.62	12.90±1.76*	8.63±0.71	6.89±0.85	2.57±0.47	2.61±0.48
OV/TV (%)	0.032±0.009	0.113±0.035*	0.083±0.036	0.057±0.019	0.017±0.004	0.022±0.003
OS/BS (%)	2.14±0.73	6.38±1.27*	3.84±1.11	3.57±1.12	1.23±0.27	1.46±0.25
O.Th (μm)	2.78±0.30	4.05±0.47*	3.06±0.20	2.68±0.16	2.39±0.22	2.75±0.39
Oc.S/BS (%)	4.60±0.21	7.63±1.31*	1.61±0.41	5.21±0.96*	1.02±0.32	2.08±0.36*
N.Oc/T.Ar (/mm^2)	9.68±0.93	10.50±1.04	3.52±0.91	11.54±2.22*	2.30±0.69	4.38±0.88
N.Oc/B.Pm (/mm)	1.60±0.08	2.65±0.40*	0.57±0.14	1.88±0.33*	0.39±0.12	0.72±0.13
ES/BS (%)	1.66±0.15	2.69±0.52	0.77±0.32	1.47±0.39	0.38±0.07	0.75±0.14*

Results are mean ± SEM
*$p<0.05$ compared with corresponding WT, unpaired *t* test

Table S4. Histomorphometric analysis of tibiae from 6-week-old female W^{sh}/W^{sh} mice and control littermates

Parameters	6-week-old	
	WT (n=7)	W^{sh}/W^{sh} (n=7)
BV/TV (%)	5.50±0.80	5.37±0.92
Tb.Th (μm)	30.33±2.03	28.36±1.93
Tb.N (/mm)	1.76±0.19	1.89±0.29
Tb.Sp (μm)	584±77	620±139
MS/BS (%)	29.37±1.81	28.51±1.99
MAR (μm/day)	1.75±0.12	2.55±0.16*
BFR/BS ($\mu m^3/\mu m^2$/year)	190±24	266±25*
BFR/BV (%/year)	1232±139	1931±136*
BFR/TV (%/year)	66±9	94±13
Ob.S/BS (%)	12.87±1.34	20.20±1.74*
N.Ob/T.Ar (/mm^2)	30.61±3.15	58.51±6.09*
N.Ob/B.Pm (/mm)	9.09±0.95	16.69±1.69*
OV/TV (%)	0.050±0.010	0.090±0.014*
OS/BS (%)	5.65±1.10	8.47±1.34
O.Th (μm)	2.67±0.16	3.26±0.30
Oc.S/BS (%)	5.18±0.68	8.46±1.09*
N.Oc/T.Ar (/mm^2)	6.35±1.10	10.55±1.03*
N.Oc/B.Pm (/mm)	1.82±0.27	3.16±0.46*
ES/BS (%)	2.28±0.49	4.51±1.02

Results are mean ± SEM

*p<0.05 compared with WT, unpaired *t* test

Table S5. Sequences of oligonucleotides for qPCR analysis

Gene	Forward primer	Reverse primer
Osteocalcin	GCTGCCCTAAAGCCAAACTCT	AGAGGACAGGGAGGATCAAGTTC
Osterix	CCCTTCTCAAGCACCAATGG	AAGGGTGGGTAGTCATTTGCATA
Bsp	TGGCGACACTTACCCAGCTT	CCATGCCCCTTGTAGTAGCTGTA
Alp	CTTGACTGTGGTTACTGCTGATCA	GTATCCACCGAATGTGAAAACGT
Type I collagen	CCCAAGGAAAAGAAGCACGTC	ACATTAGGCGCAGGAAGGTCA
Osteopontin	CTCCAATCGTCCCTACAGTCG	CCAAGCTATCACCTCGGCC
Runx2	AGTCCCAACTTCCTGTGCTCC	CGGTAACCACAGTCCCATCTG
Sost	ATCATTTCCAGACACCTCTTAC	ATGTGCTTCTGTTACAAACGCTC
Dmp1	ATGACTGTCAGGACGGCTAC	AGTTATAGTGAACTCTCTAC
RANKL	CAAGCTCCGAGCTGGTGAAG	CCTGAACTTTGAAAGCCCCA
OPG	AAGAGCAAACCTTCCAGCTGC	CACGCTGCTTTCACAGAGGTC
M-CSF	ACCTGTTTCCCAAGAAGAGAGCCT	AGCTGTCAACACAAGCAGCCAAAG
Calcitonin receptor (CTR)	GTGCTCCTCGGGCTGTAGC	GAGGATTCCGTGGTTCCTGAT
c-Fms	TGGCATCTGGCTTAAGGTGAA	GAATCCGCACCAGCTTGCTA
Nfatc1	AGGCTGGTCTTCCGAGTTCA	ACCGCTGGGAACACTCGAT
Ctsk	AGGCATTGACTCTGAAGATGCT	TCCCCACAGGAATCTCTCTG
RANK	ATGAGTACACGGACCGGCC	GCTGGATTAGGAGCAGTGAACC
TRAP	GATCCCTCTGTGCGACATCA	CCAGGGAGTCCTCAGATCCA
Efnb2	TCTGTGTCATCGGTTGGCTACGTT	ACAGACGCACAGGACACTTCTCAA
Sphk1	TGAGGTGGTGAATGGGCTAATGGA	AACAGCAGTGTGCAGTTGATGAGC
Sphk2	TGGGCTGTCCTTCAACCTCATACA	AGTGACAATGCCTTCCCACTCACT
BMP6	AGAAGGGCACTCTTTCAGGTTCCA	TCACACCACCGAGAGTCAACACAA
Cthrc1	CCCATCGAAGCCATCATCTATC	CAATCCCTTCACAGAGTCCTTC
Sema4D	TCCGCCTTGACTGTCCATGAAAGA	ACACGGTGAGCGAGAAACAAATGC
Wnt10b	AGGCTTCTCCTTCCGTTCAGTTGT	ATTCCCACCCTTCCTGCTGAAGAA
GAPDH	TGCACCACCAACTGCTTAG	GGATGCAGGGATGATGTTC
